# The Mauve Stinger *Pelagia noctiluca* (Forsskål, 1775). Distribution, Ecology, Toxicity and Epidemiology of Stings. A Review

**DOI:** 10.3390/md20080025

**Published:** 2008-09-04

**Authors:** Gian Luigi Mariottini, Elisabetta Giacco, Luigi Pane

**Affiliations:** 1 Dipartimento di Biologia, Università di Genova, Viale Benedetto XV 5, I-16132, Genova, Italy E-mails: Gian.Luigi.Mariottini@unige.it; Elisabetta.Giacco@unige.it; 2 Dipartimento di Biologia, Università di Genova, Viale Benedetto XV 5, I-16132, Genova, Italy E-mail: pane@unige.it

**Keywords:** Jellyfish, Pelagia noctiluca, venom, nematocysts, distribution, ecology

## Abstract

The toxicity of Cnidaria is a subject of concern due to its influence on humans. In particular, jellyfish blooms can highly affect human economical activities, such as bathing, fishery, tourism, etc., as well as the public health. Stinging structures of Cnidaria (nematocysts) produce remarkable effects on human skin, such as erythema, swelling, burning and vesicles, and at times further severe dermonecrotic, cardio- and neurotoxic effects, which are particularly dangerous in sensitive subjects. In several zones the toxicity of jellyfish is a very important health problem, thus it has stimulated the research on these organisms; to date toxicological research on Cnidarian venoms in the Mediterranean region is not well developed due to the weak poisonousness of venoms of jellyfish and anemones living in this area. In spite of this, during last decades several problems were also caused in the Mediterranean by stinging consequent to Cnidarian blooms mainly caused by *Pelagia noctiluca* (Forsskål, 1775) which is known to be the most venomous Mediterranean jellyfish. This paper reviews the knowledge on this jellyfish species, particularly considering its occurrence and toxicity.

## *Pelagia noctiluca*: Distribution and Blooms

The mauve stinger *Pelagia noctiluca* (Cnidaria: Scyphozoa) is a small pelagic jellyfish generally pink-, mauve- or light brown-coloured, with a phosphorescent bell measuring 3 to 12 cm in diameter in adult specimens, whose edge is provided with lappets and tentacles; in this species the nematocysts stud the tentacles, the oral arms, as well as the upper surface of the bell [1]. *Pelagia noctiluca* has direct development, so its cycle do not comprise the benthic scyphistoma stage.

This species has a wide distribution; in general, it is indicated as typical of warm waters, but due to currents it can enter temperate and cold seas, thus it can be found in tropical zones as well as in colder areas, such as in the north Atlantic and in the north Pacific; it seems to not go over 42°S and is vertically distributed mostly between 150 m depth and the surface [2], but during the day it can also be found between 300 and 500 m, with a maximum of 1,400 m [3].

*Pelagia noctiluca* was collected in intertidal zones of the Pacific Ocean along the Californian coast [4] and is widely distributed in the Mediterranean Sea and in some zones of the Atlantic Ocean. In Atlantic waters it is frequent along the Southern Atlantic French coast in summer [5]; it was observed repeatedly in swarms around the British Islands [6], and in the North Sea and also, although infrequently, off the Dutch coast [7, 8]. Strandings of *Pelagia noctiluca* were observed in 1988 on beaches of the western coast of Ireland [9]. The occurrence of *Pelagia noctiluca* in the Mid Atlantic Sea, West to Ireland, was recorded recently [10]. This jellyfish is not considered a normal resident of the North Sea and small records were made during ‘70s off the Scottish north and east coasts and in the area of Shetland and west of the Norwegian Deep; in spite of this, in 1982 *Pelagia noctiluca* were more abundant than usual, presumptively carried in by currents [11].

In the Mediterranean Sea swarms of *Pelagia noctiluca* occur usually in pelagic waters from March to May; in summer months, from June and August, isolated big specimens can also appear [12]. In the Southwestern Mediterranean this Cnidarian was indicated as the most frequent jellyfish along Tunisian coasts, mostly in autumn and winter; in this area the presence of *Pelagia noctiluca* depends on local and particular winds and currents [13]. Anyhow, this species was signalized as uncommon along Tunisian coasts [14, 15] and in front of Algeria [16].

In the Mediterranean region the studies on jellyfish had an increase during late 70’s and early 80’s of the past century, when all the Mediterranean basin was affected by an abnormal proliferation of such organisms [17, 18]. This proliferation was mainly due to *Pelagia noctiluca*, which from the irritant/toxic point of view, is also the most important Mediterranean jellyfish. In that years the proliferation of other species, such as *Rhizostoma pulmo*, *Cotylorhiza tuberculata*, *Aurelia aurita* and *Chrysaora hysoscella*, was recorded at a lesser extent and caused less health problems because of their scant poisonousness.

The *Pelagia noctiluca* bloom started in the Eastern Basin and in the Adriatic Sea (the latter was overall the most interested by the phenomenon) and subsequently spread to the Western Basin, although with less intensity. The main period concerned was 1981–1984; then, as suddenly it arose, the phenomenon finished quickly, even though sporadic proliferations were recorded in subsequent years.

In the Eastern Mediterranean the studies concerning the distribution of *Pelagia noctiluca* and of other jellyfish were carried out mainly during summer [19]; some scientists performed research on the occurrence of this jellyfish in Greek and Egyptian waters [20], along Turkey [21, 22] and Lebanese coasts [23], and lessepsian jellyfish species coming from the Red Sea were observed in Israeli waters [24, 25]. Along Maltese coasts the studies on the distribution of *Pelagia noctiluca* were carried out from 1980 to 1986 [26].

In the Adriatic Sea *Pelagia noctiluca* caused several problems to bathers and sea-workers and its distribution was studied along Croat coasts during the summer of 1983 [27] and seasonally in 1985 [28], and along Italian coasts during the period 1976–1986, mainly during the summer months, when its occurrence and seasonal variations were related with the variations of environmental factors [20, 29–32]. The influence of water temperature on swimming behaviour of *Pelagia noctiluca* was emphasized [33]; this factor could account for coastal aggregations occurring in the Adriatic coasts in summer. After research performed from 1976 to 1983, according to [34] the highest densities of *Pelagia noctiluca* in the Adriatic Sea were recorded in pelagic waters with high salinity and low nutrients. It was asserted that the dense aggregations of this organism in coastal shallow waters were wind- current- and tide-caused [35].

In the Tyrrhenian Sea and in the Ligurian Sea the consequences of the bloom were remarkably lower than in other Mediterranean zones; nevertheless, notable alterations in comparison with the normal situation were recorded everywhere from 1982 to 1988 [20, 36–42]. Furthermore, in some receptive coastal zones, such as in the Spotorno Bay (Western Ligurian Riviera), the proliferation of *Pelagia noctiluca* was evident and reached a peak during some periods (September 1984 – January 1985) with no apparent regard to the season [43]. Archived research containing data on the occurrence of *Pelagia noctiluca* during a 200 year-period showed that in the zone of Villefranche-sur-Mer (western Ligurian Sea) there were ‘years with *Pelagia noctiluca*’ and ‘years without *Pelagia noctiluca*’ every about 12 years; this could indicate the cyclic nature of the phenomenon [44]. During the ‘bloom years’, and particularly during the summer of 1983, considerable proliferation of *Pelagia noctiluca* was recorded also in Sicily and Sardinia Channels [20].

On the whole, salinity was indicated to exert an influence on jellyfish behaviour and distribution as evidenced in field observations [45]. An univocal explanation of the jellyfish bloom and of the subsequent jellyfish decrease was not supplied and several scientists provided different explanations for the blooms, but it seemed acceptable that neither hydrologic changes (as water circulation didn't show clear alterations during last decades) nor pollutant inputs supported jellyfish proliferation. It was suggested that coastal aggregation of jellyfish could be due to wind action [26], to natural [41] or cyclic [42] fluctuations of jellyfish populations or to water movements [46]. Furthermore, a relationship between jellyfish proliferation and environmental factors [23, 28] or water pollution [47, 48] was suggested. Also ‘hormesis’, which involves stimulation of growth by low concentrations of toxicants [49] and evolution processes connected to the appearance of favourable mutations [50] were suggested to induce the proliferation. Recently, the cumulative effects of man-caused and climate changes has been indicated to be involved in the mechanisms which can promote the increasing of jellyfish occurrence [51, 52]. In particular, it was stated that as Cnidaria feed high on marine food chains and therefore they can compete with fishes for food, massive removal of top-predator fishes by commercial fishing efforts could open up food resources for jellyfish [51]. In addition, models indicated that the predicted pH decrease in oceans with rising CO_2_ could induce long-term jellyfish increase over the next 100 years [52].

Jellyfish blooms have also stimulated research on the biological, ecological and chemical aspects of *Pelagia noctiluca*, which has been carried out in order to evaluate its role in the marine ecosystem and in the food web. The biological cycle of *Pelagia noctiluca* is annual [3]; for this reason it shows high natural mortality, typical of short life-cycle species [32]. Its reproductive period was also extensively studied and it was observed that *Pelagia noctiluca* reproduces throughout the year [53]. In studies concerning nutrition the gastrovascular content was examined and the diet of this organism was shown to be composed mainly (around 90%) by cladocerans and copepods [54]; other indications about the behaviour and the trophic ecology of *Pelagia noctiluca* were provided [55], observing that at 16–19°C *Pelagia noctiluca* maintenance and growth requires approximately 3 mg C/day indicating a substantial predatory impact of jellyfish when they are in aggregations.

In studies concerning the lipid composition of *Pelagia noctiluca* [56] it was observed that total lipids are 0.19 % of wet weight; within lipids these Authors found 73.8 % of neutral lipids and 26.2 % of phospholipids. Among neutral lipids mainly sterol esters (11.3 %), triglycerides (20.7 %), free fatty acids (56.6 %) and sterols (7.6 %) occur. Among phospholipids, phosphatidylcholine was particularly abundant (36 % was recorded). A high amount of free fatty acids (41.7 %) was found in the total lipid fraction; these results were comparable to data obtained from other jellyfish (*Rhizostoma pulmo* and *Cotylorhiza tuberculata*) collected in the Ligurian Sea [57]. Proteins in *Pelagia noctiluca* range from 10.9% to 19.8% of dry weight and lipids, expressed as percent of dry weight, range from 1.3% to 2.9%; only 0.2–0.3% of dry weight consisted of phosphorus [58]. Preliminary results of HPLC analyses on *Pelagia noctiluca* crude extracts partially separated by gradient density showed that a noticeable amount of the extract was of protein nature; this result was also confirmed by protein analyses [59].

Analyses of trace elements carried out on tentacles of *Pelagia noctiluca* showed that metal content does not differ significantly in different periods [60] and zinc amount was 46 μg/g dry weight in specimens collected in NW Mediterranean [61]. C and N composition of *Pelagia noctiluca* ephyrulae was experimentally evaluated as % of dry weight: values of 11.4 and 3.3 for C and N, respectively, and a C:N ratio of 4.2 were found [62]. C isotope analysis showed values of −18.51±1.12 δ^13^C‰ [63, 64]. Sphingophosphonolipid composition of *Pelagia noctiluca* was recently defined and a suite of two ceramide 2-aminoethylphosphonic acids (CAEP) was quantified at 2.0 and 1.3% of phospholipids [65]. The pH of body fluids taken from *Pelagia noctiluca* collected in the intertidal zone from Laguna Beach (California) was also performed; the results showed a pH ranging from 7.304 to 7.307 [4].

## Nematocyst Morphology

The morphology of nematocysts of *Pelagia noctiluca* was observed during early twentieth century [66, 67]; subsequently [6] three types of nematocysts were described: heterotrichous microbasic eurytele, holotrichous isorhiza and atrichous isorhiza [68]. Subsequently two predominant types of nematocysts were referred to be found on *Pelagia* tentacles: a round holotrichous isorhiza (20–25 μm diameter) containing multiple spiralled barbed nema inside and a microbasic mastigophore, approximately one-fourth the size the former [69]. On the whole, *P. noctiluca* holotrichous isorhiza are longer than similar nematocysts of other Scyphozoan medusae such as *Catostylus mosaicus* and *Phyllorhiza punctata* [70].

More recent studies showed five morphological types that have been differentiated according to the classification of Mariscal [71, 72]: heterotrichous microbasic eurytele, holotrichous isorhiza haploneme (type I), atrichous isorhiza haploneme (type I), holotrichous isorhiza haploneme (type II) and atrichous isorhiza haploneme (type II).

Subsequently, these types were better defined by ultrastructural studies [68] as follows: heterotrichous microbasic eurytele, holotrichous O-isorhiza, heterotrichous isorhiza, atrichous a-isorhiza and another type resembling the microbasic p-mastigophore.

Recently, the morphology of *Pelagia noctiluca* nematocysts was re-examined [59] and the nematocysts found were separated in three groups including different morphological types: Group 1: great-spherical nematocysts corresponding to the holotrichous isorhiza and large atrichous isorhiza, that previously were stated to be undistinguishable when the capsule is undischarged [68]; Group 2: smaller and elliptical nematocysts corresponding to the heterotrichous microbasic eurytele and the heterotrichous isorhiza [68]; Group 3: smallest elliptical nematocysts with protruded operculum, similar to group 2 nematocysts, excepting the size.

The nematocysts of *Pelagia noctiluca* can be found on umbrella, oral arms and tentacles; thus all the jellyfish is venomous and discharge can be induced, aside from encounters with the living animal, even from handling stranded or dead organisms [73].

## Nematocyst Discharge

The nematocysts of *Pelagia noctiluca* can be maintained isolated in distilled water where they retain their discharging capacity [74]. The discharge of isolated nematocysts can be induced by treatment with trypsin, while Ca^2+^ ions prevent discharge [74]. Extreme values of pH (<2 and >11) promote discharging of isolated nematocysts; furthermore, aqueous solutions at pH 1.0–3.5 cause collapse of the capsular wall of undischarged nematocysts [75]. The nematocysts of *Pelagia noctiluca* may be triggered by vinegar [76].

Nematocyst discharge in *Pelagia noctiluca*, as in other Cnidaria such as in some Anthozoa [77, 78], is a Ca^2+^-dependent process [79]. Previous treatment of oral arms with La^3+^ inhibits nematocyst discharge and a similar result was obtained by treatment with Gd^3+^, a powerful blocker of mechanosensitive ion channels; therefore, nematocyte activation seems to be Ca^2+^ dependent and Ca^2+^ permeable mechanosensitive channels were demonstrated to be involved in the activation of nematocytes [79]. The anions, such as I^−^, Cl^−^, SO_4_^2−^ play an important role to promote discharge of nematocysts. In particular, I^−^ was the most effective and discharging activity decreased by using Cl^−^ and was very low with SO_4_^2−^; from these results it was stated that ion effect is probably exerted on protein conformation of the capsular wall/fluid [80]. The hypothesis that a conformational change of a capsular protein is involved in the discharging process was confirmed also by studies which evaluated the discharging effectiveness of Hofmeister anions, in which SO_4_^2−^ showed the lowest discharging potency and SCN^−^ the highest one [81]; nematocyst discharge can be induced also by thioglycolate with reduction of –S–S– bridges [82].

## Jellyfish Stings and Epidemiology

It is well known that jellyfish stings can induce both local and general symptoms [83] and sometimes can be lethal to humans [84]. In general, though, jellyfish stings usually cause a mild local dermatitis; so serious or fatal systemic reactions are uncommon [85]. After envenomation some neuromuscular manifestations such as localised neuropathy and mononeuritis multiplex [86–88] as well as neurological manifestations such as delirium, stupor, central respiratory failure and muscular weakness have been reported [85]. Guillain-Barré syndrome was also described, presumably a consequence of an aberrant immune response [89].

*Pelagia noctiluca* stings are usually limited to the skin surface and cause only erythematous, edematous, and vesicular topical lesions [90, 91], with local pain which persists for 1–2 weeks [91], while systemic complications or cutaneous infections are infrequent [92].

Nevertheless, dramatic immediate reactions have been observed after *Pelagia noctiluca* stings, even though they are rarely severe and prolonged; the lesions appear circinate or irregularly shaped, the venom is also able to cause severe generalized allergy with bronchospasm, pruritus and postinflammatory hyperpigmentation [93]. Immediate pain, distress, occurrence of urticaria-like lesions and dyspnoea after massive stingings were reported [94]. Serious consequences may be produced by immunological and/or toxic mechanisms [95, 96]. *Pelagia noctiluca* stings can leave scars and hyperpigmentation; the persistence of pigmentation can result from tattooing of the mauve stinger pigment into the skin or from post-inflammatory events [83]. Hyperpigmentation can remain for some years after envenomation causing aestethical problems [73, 92]. It was reported that jellyfish probably identificated as *Pelagia noctiluca* caused recurrent and more severe cutaneous eruptions over ten days after envenomation [97].

Stinging by *Pelagia noctiluca* can provoke relapse of the eruption after some years, also without further contact with jellyfish; thus was hypothesized the venom could react with dermal collagen and produce an active antigen which stimulates the immunological response [91]. Cross-reactions against venom of *Physalia physalis* was observed in patients showing significant titers of IgG against crude extract of nematocysts of *Pelagia noctiluca* [98]. Anyhow, the seriousness of envenomation is due to the eventual allergic characteristics of the patient or to previous envenomations [73].

The jellyfish bloom gave rise to epidemiological studies, which were carried out mostly in the Adriatic area [99–101] and in the Eastern Mediterranean region [102]. In the Northern Adriatic during 1983 67 subjects suffered from jellyfish stings with pain, swelling and erythema along the littoral of Portorož (Slovenia); most of injuries were local, on arms and chest, and limited to small areas and systemic reactions were observed in three cases [99]. In the zone of Trieste (Italy) from 1978 to 1983 127 subjects come into contact with *Pelagia noctiluca* and suffered from skin and systemic injuries mainly with local symptoms; only five cases, whose atopy was ascertained through family and personal history, had more severe general symptoms [103]. In Pula (Croatia) 52% of bathers were stung by jellyfish during summer 1978, some of them several times [100, 101]. In Greek waters 762 subjects, mainly adults, required medical advice from 1981 to 1984, particularly in July and August during the swimming period; 82.8% of the cases were observed in the coastal area of Attica and most of them (92.3%) suffered local symptoms (redness, pain, itching, burning, vesicles), 7.7% had general symptoms (dizziness, vomiting, fall of blood pressure and diarrhea, one case shock) [102]. On the whole in Adriatic coastal zones bathing was significantly influenced by the bloom [101]. Otherwise, a scarce incidence of dermatitis caused by *Pelagia noctiluca* was emphasized in the Ligurian region where the observed cases were of scarce clinical relevance: the available data, concerning only stung children from 1984 to 1989 indicate only 20 cases were observed and none of them was hospitalized [40, 104].

## Nematocyst Isolation and Toxicity of the Venom

The problem of the separation of nematocysts from tissue material has been dealt with by several scientists. As already reported for other Cnidaria [72, 104–110], in the case of *Pelagia noctiluca* the toxicity is not exclusively due to nematocysts, but is also ascribable to tissue components [111].

An attempt to obtain pure suspensions of nematocysts from *Pelagia noctiluca* was made by soaking of tissues in distilled water and subsequent centrifugations in saccarose solution at 4°C with lyophylisation of the obtained undischarged nematocysts which can thus maintain their discharging capacity when put at room temperature [112].

Recent laboratory data showed the nematocysts of *Pelagia noctiluca* can be separated by centrifugation using the discontinuous density gradient of Percoll [59]. Furthermore, nematocysts were recently isolated by chemical (treating them with SCN^−^) and physical methods (heat dissociation), and it was observed that heat dissociation promotes the release of nematocysts from tissues, but causes damage of nematocytes, so, it is scarcely useful; on the other hand, treatment of tentacles with SCN^−^ yields 90% of intact nematocytes [113]. In addition, the preservation of the nematocysts of *Pelagia noctiluca* depends on maintenance method and particularly on temperature (freezing) and pH (neutral values are optimal) [114].

In other studies the nematocysts from marginal tentacles of *Pelagia noctiluca* were isolated and partially purified; then lyophilised fractions of homogenate preparations of nematocysts were fractionated on Sephadex G-75 columns, the molecular weight was determined and fractions were tested to evaluate the effect on heart activity of rats and on neuromuscular activity of frogs. The results showed an evident toxic activity of unfractioned pools on neuromuscular synapses and less myocardial effects with only few variations of cardiac frequency [72].

Preparations of intact nematocysts were tested on hairless mice and on human skin to evaluate their irritant effects: mice were seen to develop erytema and papules after contact with the preparation; intradermal injection produced erytema, oedema, leukocyte infiltrate and nodular lesions with central necrosis [115]. On human skin the most irritant effects were obtained with the scratch test which showed the occurrence of erytema after 30 minutes accompanied by pruritus in less than 50% of cases; these symptoms decreased progressively after 48 and 72 hours [90].

A partial purification of a cardiotoxin in crude *Pelagia* venom was obtained using anti-*Chrysaora* or anti-*Physalia* monoclonal antibody–Sepharose columns. In these experiments protein bands with molecular weights of 54,000, 92,000, 130,000 and 150,000 were revealed and was also reported that both crude and partially purified *Pelagia* venom contained active fractions against cultured chick embryo cardiocytes [116].

The venom of *Pelagia noctiluca* is of protein nature and contains peptides; it is antigenic and possesses dermonecrotic and hemolytic properties; electrophoretical analyses recognized eight different fractions, distinguished by molecular mass [101].

The capsule fluid and the capsule wall of the nematocysts of *Pelagia noctiluca* were widely studied from the point of view of their protein content: it was observed that in both structures glutamic acid is the most frequent aminoacid (80% in the proteins of the capsule fluid and 90% in that of the capsule wall); these glutamate-rich proteins are probably stabilized by Ca^2+^; discharging agents (lyotropic anions, proteolytic enzymes, Ca^2+^ chelating agents) could act on capsule wall proteins inducing their conformational change [117].

As referred above, a cross-reactivity between *Pelagia* venom and monoclonal antibodies to *Physalia* and *Chrysaora* venoms [116] was reported; this aspect was clinically verified by the release of histamine after exposure to *Chrysaora* venom by basophils from a patient who had clinical anaphylaxis after a *Pelagia* sting [96]. So, also clinical evidences could demonstrate the cross-reactivity between the venoms of these jellyfish; *Pelagia* venom has got more antigenic potential for man than several other jellyfish venoms [69].

The cytotoxic properties of *Pelagia noctiluca* crude venom have been experimentally assessed by short-term [111] and long-term [104] tests on cultured cells by trypan blue dye exclusion, neutral red, colony forming efficiency and genotoxicity assay. Crude *P. noctiluca* venom highly affected cultured cells, producing severe survival decrease but, despite its well-known *in vivo* irritating properties, it showed lower effects in comparison to that evidenced by venoms of other jellyfish and anemones apparently less venomous *in vivo* [107–110]. Anyhow, the venom of *Pelagia noctiluca* showed remarkable cytotoxicity, and killed all treated cells at highest tested concentration within two hours. The protein nature of venom [96] was further confirmed by the absence of effects on DNA of treated cells [111]. *Pelagia noctiluca* venom caused also an increase of ATP levels in treated cells within 1 hour of treatment and a following moderate decrease [111]; this is a strange behaviour, because in general toxicity studies record decrease of ATP in stress-exposed cells [118, 119] and organisms [120]. Also long term cell proliferation tests showed that the venom of *Pelagia noctiluca* has less effect on cells than venom of other jellyfish [104].

The hemolytic properties of the crude venom of *Pelagia noctiluca* were recently assessed on fish, chicken, rabbit and human erythrocytes; results showed a significant hemolysis of chicken and rabbit erythrocytes and a good resistance of fish ones. It was also observed that crude venom maintains its hemolytic properties even after freezing at −20°C and −80°C and lyophilization [121].

## Conclusions

### Distribution and bloom

A satisfactory explanation of the bloom of *Pelagia noctiluca* in the Mediterranean, and partly in Atlantic waters has not been provided, even though several causes have been indicated as responsible of the phenomenon; a number of scientists have supposed that it could be the result of natural cyclic fluctuations, already described in several species, correlated with environmental or trophic factors [23, 28, 36, 41, 42, 46]. On the other hand, lack of alteration in lipid content of jellyfish during [57] and after the bloom also prevented scientists from hypothesizing about the implications of food quantity and quality in supporting the phenomenon.

On the whole, outbreaks of *Pelagia noctiluca* can have an important ecological impact on plankton dynamics and on trophic relationships; this was seen particularly in the Adriatic Sea, where the bloom showed its maximum [122]; as a matter of fact, events of massive occurrence of planktonic organisms could cause alterations of community structures and functioning as well as lack of biodiversity [123]. As *Pelagia noctiluca* is a top predator and its feeding activity is exerted on several zooplankters, including eggs and larvae of nektonic and benthic organisms, this impact could have affected remarkably prey populations and consequently caused lower production [122]. In this connection, from the biological and ecological point of view the chemical analyses carried out on *Pelagia noctiluca* specimens [62, 64, 65] are important in order to evaluate the transfer of energy to high trophic levels as well as the amount of C and N available for microorganisms in consequence of the decomposition, which becomes more intense during outbreaks.

### Nematocyst morphology and discharge

The nematocysts of *Pelagia noctiluca* can be found on the umbrella, oral arms and tentacles; they have been classified into five morphological types [68, 72] and recently they were separated into three groups [59]. It is known that the activation of nematocysts is calcium dependent and discharge is promoted by anions [75] and inhibited by lantanium and gadolinium [79]. Furthermore, the venom of this jellyfish is cytotoxic [111] and studies performed on cell cultures, that are an efficient alternative method to the utilization of living organisms, showed also cell growth decrease after treatment.

At a cellular level it was suggested that Cnidarian venoms affect the plasmalemma by binding to membrane phospholipids and increasing permeability with consequent water uptake and damage of external and intracellular membranes [124]; the phospholipase activity of Cnidarian venoms, which affects cell membrane permeability and ion exchange, was also suggested [125–127].

### Toxicity and epidemiology

*Pelagia noctiluca* has not caused human fatalities, but in spite of this it can be a nuisance and a health and economical problem when it appears in huge numbers during outbreaks. As a matter of fact, the contact with all body portions – bell, tentacles and oral arms – causes in humans local pain, burning, swelling, hyperpigmentation and other local symptoms; repeated contacts with this jellyfish can produce recurrent skin eruptions, which were also observed without further stingings.

During the bloom the epidemiological studies on *Pelagia noctiluca* stings were carried out mostly both in Italian and Croatian Adriatic localities [99–101] owing to the high impact on bathers and fishermen in these zones, while data from other Mediterranean regions are sporadic [40, 102, 104]. On the whole, data concerning the impact on human health are scarce and not many subjects come under medical observation, even though presumptively several cases have not been seen in medical first-aid stations.

### Lack of knowledge

A lot remains unknown, both about the ecology and toxicity of *Pelagia noctiluca* and several aspects remain to be clarified to explain its complex biological and ecological role in the marine environment. The causes and the dynamics of blooms in relation to the variations of environmental factors, to climate and to man-caused changes are to date greatly unknown; in this connection, the alterations induced on marine food chains [51] and the consequences of CO_2_ rising on pH of oceans, which was seen to be correlated with jellyfish frequency [52], are stimulating research subjects.

From the toxicological point of view, further research is also required to better separate the nematocyst content from tissue components of *Pelagia noctiluca*, in order to characterize the toxic compounds which produce the clinical symptoms and the observed cytotoxicity *in vitro*. It should be also important to explain how venom acts against cells and to clarify the mechanisms which damage biomembranes and alter membrane permeability.

Finally, as several substances extracted from marine organisms have bioactive and pharmacological properties it should be interesting and useful to know if compounds with such characteristics occur in *Pelagia noctiluca* too.
